# The Vagus Nerve at the Interface of the Microbiota-Gut-Brain Axis

**DOI:** 10.3389/fnins.2018.00049

**Published:** 2018-02-07

**Authors:** Bruno Bonaz, Thomas Bazin, Sonia Pellissier

**Affiliations:** ^1^Division of Hepato-Gastroenterology, University Hospital, Grenoble Alpes, France; ^2^Grenoble Institute of Neurosciences, University Grenoble Alpes, Inserm U1216, Grenoble, France; ^3^Institut National de la Recherche Agronomique, Mycoplasmal and Chlamydial Infections in Humans, Univ. Bordeaux, Bordeaux, France; ^4^Department of Hepato-Gastroenterology, Bordeaux Hospital University Center, Pessac, France; ^5^LIP/PC2S, Université Grenoble Alpes, Université Savoie Mont Blanc, Grenoble, France

**Keywords:** microbiota-gut-brain axis, vagus nerve, vagus nerve stimulation, cholinergic anti-inflammatory pathway, stress

## Abstract

The microbiota, the gut, and the brain communicate through the microbiota-gut-brain axis in a bidirectional way that involves the autonomic nervous system. The vagus nerve (VN), the principal component of the parasympathetic nervous system, is a mixed nerve composed of 80% afferent and 20% efferent fibers. The VN, because of its role in interoceptive awareness, is able to sense the microbiota metabolites through its afferents, to transfer this gut information to the central nervous system where it is integrated in the central autonomic network, and then to generate an adapted or inappropriate response. A cholinergic anti-inflammatory pathway has been described through VN's fibers, which is able to dampen peripheral inflammation and to decrease intestinal permeability, thus very probably modulating microbiota composition. Stress inhibits the VN and has deleterious effects on the gastrointestinal tract and on the microbiota, and is involved in the pathophysiology of gastrointestinal disorders such as irritable bowel syndrome (IBS) and inflammatory bowel disease (IBD) which are both characterized by a dysbiosis. A low vagal tone has been described in IBD and IBS patients thus favoring peripheral inflammation. Targeting the VN, for example through VN stimulation which has anti-inflammatory properties, would be of interest to restore homeostasis in the microbiota-gut-brain axis.

## Introduction

A huge amount of data has highlighted a potential role of microbial dysbiosis in various chronic disorders (Lynch and Pedersen, 2016). The microbiota, the gut, and the brain communicate through the microbiota-gut-brain axis and a perturbation of this axis is involved in the pathophysiology of neurodegenerative disorders (Cenit et al., 2017; Kobayashi et al., 2017; Quigley, 2017). The brain and the gut communicate in a bidirectional way, through the autonomic nervous system (ANS) and the circumventricular organs (Bonaz and Bernstein, 2013). A perturbation of this axis is involved in the pathophysiology of gastrointestinal (GI) disorders such as irritable bowel syndrome (IBS) and inflammatory bowel disease (IBD) which are biopsychosocial diseases (Mulak and Bonaz, 2004; Porcelli, 2004; Bonaz and Bernstein, 2013; Oświecimska et al., 2017). The vagus nerve (VN), the principal component of the parasympathetic nervous system, is considered as the sixth sense (Zagon, 2001) because of its role in interoceptive awareness (Strigo and Craig, 2016; Smith et al., 2017). The VN is able to sense the microbiota, to transfer this gut information to the central nervous system where it is integrated, and then to generate an adapted or inappropriate response; the latter could perpetuate a pathological condition of the digestive tract or favor neurodegenerative disorders (Eisenstein, 2016; Tse, 2017). A dysbiosis is observed in IBS and IBD. However, in the context of this axis, the question is whether it is a cause or a consequence of an abnormal gut-brain processing. Stress, either interoceptive or exteroceptive, is involved in the pathophysiology of IBS and IBD and can modify the gut microbiota (O'Mahony et al., 2009; Konturek et al., 2011; Bonaz, 2013; Barbara et al., 2014). Stress stimulates the sympathetic nervous system while inhibiting the VN (Porges, 1995; Sahar et al., 2001). The VN, a mixed nerve with anti-inflammatory properties both through its afferent and efferent fibers, is at the interface of the brain-gut axis (Bonaz et al., 2016a,b). An abnormal vagal tone is described in IBS and IBD (Pellissier et al., 2010, 2014). Targeting the VN could restore homeostasis in such diseases. In particular, VN stimulation (VNS), approved for the treatment of depression and epilepsy (Ben-Menachem, 2001; Bonaz et al., 2013) and for its anti-inflammatory properties (Borovikova et al., 2000a,b; Pavlov et al., 2003), should be of interest.

## How the microbiota communicates with the brain?

**Figure 1:**
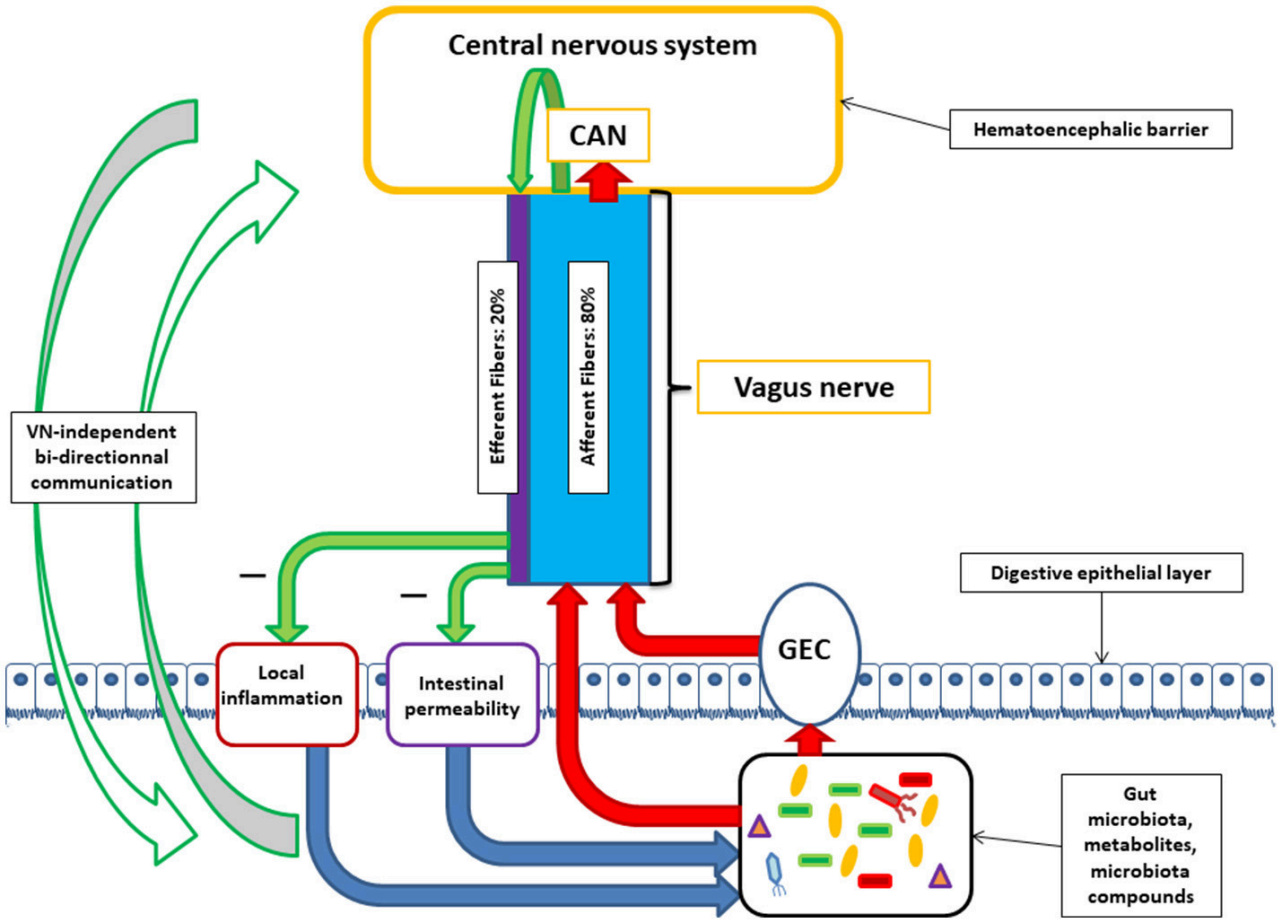
Communication between the central nervous system and the microbiota through the vagus nerve (VN). VN afferent fibers can be stimulated by microbiota components either directly or indirectly via gut endocrine cells (GEC). VN afferent fibers exert stimuli on the central nervous system via the central autonomic network (CAN). VN afferent fibers are able to stimulate efferent fibers through the inflammatory reflex. VN efferent fibers can reduce digestive inflammation and reduce intestinal permeability by tight junction reinforcement. These actions of vagal efferent fibers can indirectly modulate microbiota composition. Alongside with brain-VN-microbiota axis exists bi-directional communication by various ways.

The human gut contains 10^13^–10^14^ microorganisms, much more than the cells of our organism, and 100 times more genes than our genome. The weight of the microbiota is about 1 kg in the adult. The vast majority of bacteria resides in the colon. In healthy adults, two bacterial phyla, Bacteroidetes and Firmicutes, dominate bacterial composition, with smaller amounts of Actinobacteria, Proteobacteria, and Verrucomicrobia. Gut microbiota also contains methanogenic archae, eucaryotes (mainly yeasts), and numerous phages (Eckburg et al., 2005; Reyes et al., 2010). The communication between the brain and the microbiota is bidirectional, through multiple pathways: neural through the VN and/or spinal cord, endocrine (through the hypothalamic pituitary adrenal, HPA, axis), immune (cytokines), and metabolic [short chain fatty acids, (SCFAs), tryptophan…] (Cryan and Dinan, 2012; Brookes et al., 2013; Perez-Burgos et al., 2015; Forsythe et al., 2016; Sarkar et al., 2016). Neuroactive compounds are released by bacteria such as γ-aminobutyric acid (GABA), serotonin, dopamine, acetylcholine (ACh), and essentially act locally on the enteric nervous system i.e., the gut brain (Lyte, 2011; Sarkar et al., 2016). Some of these compounds reach the big brain via blood and circumventricular organs or through the VN. In this minireview, we will focus on the involvement of the VN at the interface of the microbiota-gut-brain axis (Figure 1).

## The vagus nerve in the microbiota-gut-brain axis

The VN contains 80 and 20% of afferent and efferent fibers respectively (Agostoni et al., 1957) and innervates all the digestive tract for some authors (Delmas and Laux, 1933) or until the left colonic flexure for others (Netter, 1989). In both cases, an interaction between VN fibers and microbiota-rich colonic mucosa is possible.

### Gut to brain interactions

Vagal afferent fibers are distributed to all the layers of the digestive wall but do not cross the epithelial layer (Wang and Powley, 2007) so that they are not in direct contact with the gut luminal microbiota. Consequently, these fibers can sense only indirectly microbiota signals, through the diffusion of bacterial compounds or metabolites, or thanks to other cells located in the epithelium that relay luminal signals. Interactions between gut endocrine cells and vagal afferents are at the interface of gut chemosensing (Raybould, 2010). Enteroendocrine cells (EECs), 1% of intestinal epithelial cells, release their content in presence of luminal carbohydrate, triglycerides, and protein and modulate GI functions such as motility, secretion, and food intake (Näslund and Hellström, 2007; Gunawardene et al., 2011; Wu et al., 2013). EECs interact with vagal afferents either directly through the release of serotonin (5-hydroxytryptamine, 5-HT) activating 5-HT3 receptors located on vagal afferent fibers (Li et al., 2000) or gut hormones such as cholecystokinin (CCK), glucagon-like peptide-1, peptide YY targeting the brain through vagal afferents which express receptors for these anorexigenic or orexigenic (ghrelin, orexin) hormones (Strader and Woods, 2005). Brain pathways activated by these hormones have been mapped using *c-fos* expression as a marker of neuronal activation (Bonaz et al., 1993a,b). EECs detect signals from microbiota through toll-like receptors (TLR) which recognize bacterial products such as lipopolysaccharides (LPS) and others (Abreu et al., 2005), and are expressed by EECs (Bogunovic et al., 2007), or receptors for microbiota metabolites such as SCFAs (Samuel et al., 2008). Thus, EECs are key players in the detection of luminal bacterial content and bacterial products that can regulate GI motility, secretion, food intake, through their indirect effect on vagal afferent fibers.

In addition with cell-mediated sensing, VN can sense microbiota signals through direct mechanisms. For instance, SCFAs produced by the microbiota activate vagal afferent fibers by different mechanisms depending on the compound: while oleate, a long fatty acid, acts on vagal afferents through a CCK-mediated mechanism, butyrate, a short fatty acid, has a direct effect on afferent terminals (Lal et al., 2001).

In addition, TLR4 are expressed on vagal afferent fibers (Goehler et al., 1999) and these fibers could sense bacterial products such as LPS to activate the brain. LPS also activate directly vagal afferent fibers at the level of the nodose ganglia (Hosoi et al., 2005) thus explaining that subdiaphragmatic vagotomy does not completely block brain mediated behavioral and neural effects of peripheral LPS or interleukin-1beta (Schwartz et al., 1997).

**Figure 2:**
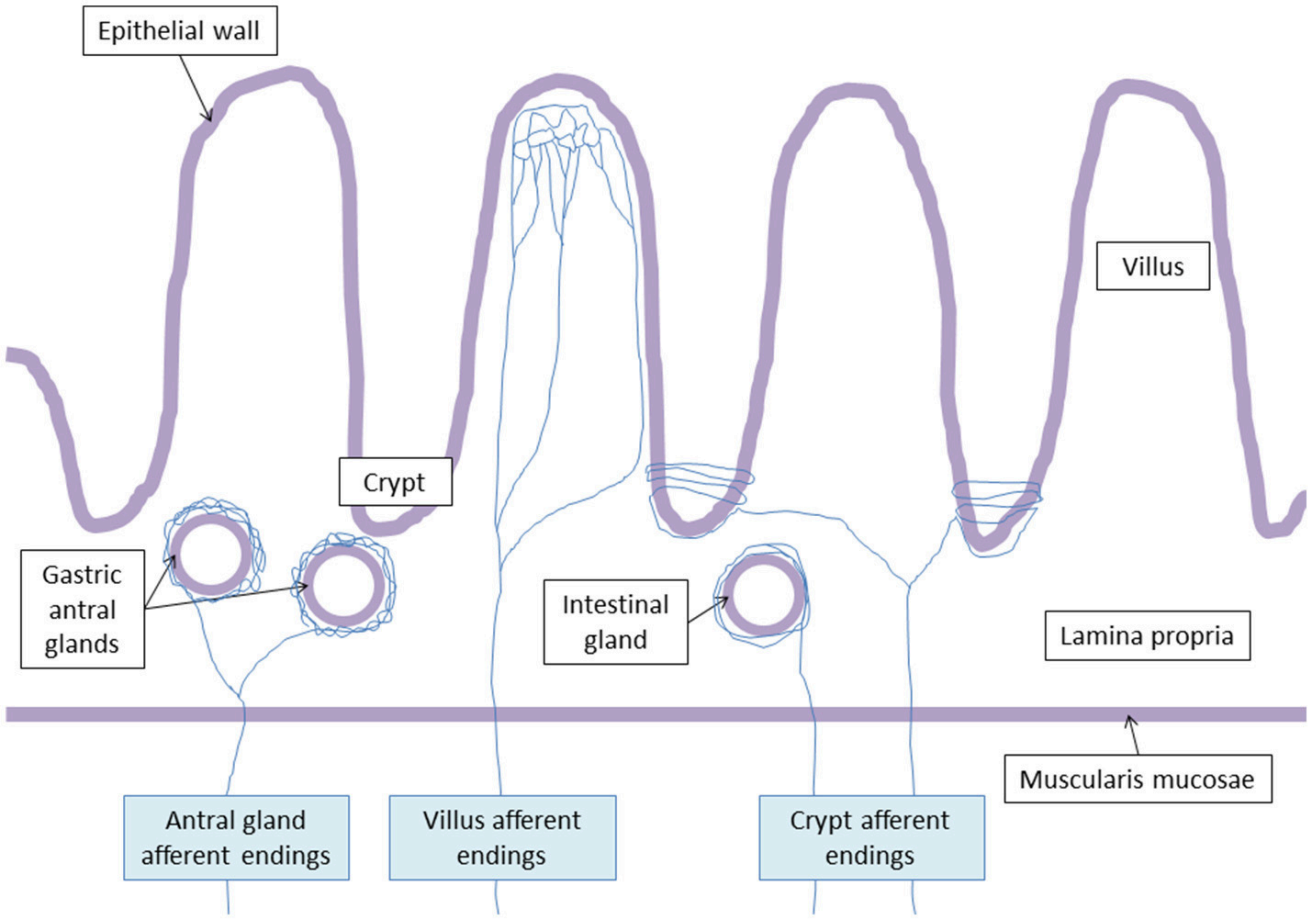
Vagal terminal afferent endings. Antral gland afferent endings begin to divide at the level of the muscularis mucosae, and surrond gastric antral glands creating arbors. Villus afferent endings divide at the basal pole of the crypts, and ramify repeatedly at the apical half of the villus. Crypt afferent endings divide at the basal pole of the crypts, and collaterals encircle multiple time the crypts or the intestinal glands.

Powley et al. (2011) have shown that vagal afferent endings are divided into three subtypes: villus afferent endings are distributed at the apical tips of intestinal villi, immediately below the epithelial wall while other types of afferent endings are independently distributed either around the intestinal glands or crypts (crypt afferent endings), immediately below the crypt-villus junction, or along the gastric antral glands (antral gland afferent endings) and forms terminal concentrations immediately below the luminal epithelial wall (Figure 2). These endings are both chemo- and mechanosensitive. Blackshaw et al. (Blackshaw and Grundy, 1993a,b) have recorded single afferent fibers with receptive fields in the mucosa of the upper GI tract from the cervical VN of urethane anesthetized ferrets. Single chemosensitive vagal afferent units supplying the gut respond to most luminal molecules by increasing their firing rate. They observed a broad sensitivity of mucosal afferent fibers to luminal chemical and mechanical stimuli. Mucosal endings were stimulated by 5-HT acting directly on afferent endings via 5-HT3 receptors.

Vagal chemoreceptors are most likely involved in the communication between the microbiota and the brain by sensing SCFAs and/or gut hormones (Raybould, 2010). Indeed, intraduodenal injection of *Lactobacillus johnsonii* enhanced gastric VN activity (Tanida et al., 2005). Healthy mice chronically treated with *Lactobacillus rhamnosus* (JB-1) presented GABA brain expression modifications which increased in the cingulate cortex and decreased in the hippocampus, amygdala, and locus coeruleus. These animals had also reduced stress-induced corticosterone and anxiety- and depression-related behavior. These effects were not observed after vagotomy. Hence, *L. rhamnosus* have potential therapeutic indications in stress-related disorders pointing out the vagally-mediated microbiota effect on mood (Bravo et al., 2011).

Using an *ex vivo* intestinal jejunal segment mesenteric nerve recording preparation, Perez-Burgos et al. (2013) have shown that administration of *L. johnsonii* in the lumen of this segment induced an increase in the firing rate of vagal afferents prevented by prior subdiaphragmatic vagotomy. As electrical stimulation of vagal afferent fibers modifies brain concentration of serotonin, GABA, and glutamate (Ressler and Mayberg, 2007) and is used in the treatment of drug resistant epilepsy and depression (Bonaz et al., 2013), this study suggests that probiotic-mediated VN activation could have beneficial effects in these diseases. However, as no *in vivo* experiment has been yet conducted, VN afferent fiber direct stimulation by digestive microbiota is still hypothetical.

The activation of vagal afferent fibers by microorganisms was highlighted indirectly by the group of Mark Lyte (Gaykema et al., 2004) using *c-fos* as a marker of neuronal activation to map brain pathways activated by oral administration of *Campylobacter jejuni* at subclinical doses in mice influencing behavior and brain functions. Brain activation was observed in the nucleus tractus solitarius (NTS), the first entrance of vagal afferents in the brain, as well as in the widespread projections of the NTS such as the parabrachial nucleus, PVH, amygdala which are part of the central autonomic network (CAN) (Gaykema et al., 2004; Goehler et al., 2005, 2008). The CAN is involved in the autonomic, endocrine, cognitive, and behavioral response to interoceptive and exteroceptive stimuli (Benarroch, 1993) and appears as the relay of vagal afferents, in particular of visceral origin, in the central nervous system (Bonaz and Bernstein, 2013).

In addition, Bercik et al. (2011) have shown that *Bifidobacterium longum* has anxiolytic effects during non-infectious, chronic, low-grade gut inflammation induced by dextran sodium sulfate administration. This effect required vagal integrity, and was probably mediated by vagal pathways originating at the level of the enteric nervous system.

Bellono et al. (2017) have recently detailed the mechanisms involved in the communication between gut epithelial cells, particularly EECs, and the neural system by generating murine intestinal organoids where EECs were genetically tagged with green fluorescence protein to characterize their physiologic, pharmacologic, and molecular functions through electrophysiological analysis. They showed that EECs are excitable and express voltage-gated sodium and calcium channels. They screened 30 compounds present in the gut such as microbial products, irritants and inflammatory agents, and neurotransmitters. They showed that only allyl isothiocyanate, a mustard plant component that induces visceral inflammatory pain, isovalertate, a volatile fatty acid fermentation product produced by gut microbiota associated with GI disorders, and the catecholamines (dopamine, epinephrine, and norepinephrine), involved in GI stress, specifically activate EECs. Isobutyrate and butyrate elicited small but consistent responses. They showed that EECs express sensory receptors, which detect and transduce specific signals, such as transient receptor potential cation channel A1, an irritant receptor for allylisothiocyanate, the olfactory receptor 558 which serves as a microbial metabolite sensor activated by isovalerate, and an α2A adrenoreceptor-TRPC4 channel signaling cascade that detects stress response-related catecholamines. Using imaging of intestinal preparations, they showed that many 5-HT3R-expressing fibers, colocalized with synaptic markers thus of neural origin, innervate gut epithelium and make close contacts with EECs. By recording mechanosensory nerve fibers from an *ex vivo* colonic preparation, they showed that noradrenaline or isovalerate applicated to the epithelium induced responses in the sensory neurons dependent on EEC-mediated transduction through 5-HT3 receptors. They concluded that EECs are polymodal chemosensors that integrate extrinsic and intrinsic signals within the gut and convey this information to the nervous system. These data have potential therapeutic implications in the domain of IBS and IBD. In this study, they did not characterize nerve fibers in close contact with EECs but sympathetic and vagal sensory fibers communicate with EECs (Williams et al., 2016). Serotonin released by EECs may also act on enteric neurons (Veiga-Fernandes and Mucida, 2016). Together, communication between bacterial products and the VN seems possible, but does not necessarily happen permanently during physiological conditions.

Non-vagal pathways are also involved in the microbiota-gut-brain axis (Mayer et al., 2015). For instance, van der Kleij et al. (2008) showed the protective effects of *Lactobacillus reuteri* and *Bifidobacterium infantis* in a model of dextran sulfate sodium colitis in mice after subdiaphragmatic vagotomy. Both strains did not require the presence of an intact VN for their protective effects.

### Brain to gut interactions

Central vagal stimulation with a thyrotropin-releasing hormone analog, known to activate preganglionic neurons of the dorsal motor nucleus of the VN, activates M2 macrophages which are in close connection with gastric cholinergic myenteric neurons and have anti-inflammatory properties, and deactivates pro-inflammatory M1 macrophages in the stomach which are involved in abdominal surgery-induced gastric inflammation and gastric ileus (Yuan and Taché, 2017). However, few objective data are available on the pro- or anti-inflammatory role of M1 and M2 macrophages on intestinal permeability. M2 macrophages did not play a role in the increase of intestinal permeability observed in a model of enteric nematode infection (Notari et al., 2014). The VN is able to inhibit M1 proinflammatory macrophages (Yuan and Taché, 2017) and this anti-inflammatory effect could modify intestinal permeability and/or the gut microbiota.

Vagal afferents activate vagal efferents in an inflammatory reflex described in 2000 by the group of Tracey in a model of septic shock and called the cholinergic anti-inflammatory pathway (CAP) (Borovikova et al., 2000a,b; Martelli et al., 2016). Indeed, ACh released at the distal end of vagal efferents inhibits the release of TNFα by macrophages through their α7nicotinicACh receptors (α7nAChR) (Wang et al., 2003). In the same way, a vago-sympathetic pathway to the spleen inhibiting TNFα release by splenic macrophages has been described (Olofsson et al., 2012) and for some authors the splanchnic pathway is the efferent arm of the inflammatory reflex (Martelli et al., 2016). In addition, vagal afferents, through the widespread projections of the NTS, target the CAN which in return modulates the ANS, through descending pathways from the PVN, A5, and C1 noradrenergic and adrenergic group respectively (Bonaz et al., 2017). When targeting vagal afferents to the brain, the gut microbiota could modulate this inflammatory reflex either activating or inhibiting the VN thus being anti- or pro-inflammatory.

The intestinal epithelium is a barrier to prevent translocation of bacteria and other agents. Severe burn injuries in a rat scald model injuring 35% of the total body surface area result in intestinal barrier dysfunction due to gut ischemia and electroacupuncture has a protective role through the VN by decreasing intestinal permeability (Hu et al., 2013; Wang et al., 2015). VNS increases the expression and proper localization of tight junction proteins and decreases intestinal epithelial permeability (Zhou et al., 2013; Van Houten et al., 2015). In the same way, electroacupuncture prevents intestinal barrier dysfunction following prolonged hemorrhagic shock by decreasing intestinal permeability through a vagal anti-inflammatory mechanism (Du et al., 2013). In the same model, VNS had a protective effect, independent of the spleen but involving a cholinergic nicotinic receptor since this effect was reproduced with nicotine (Levy et al., 2013). Lipid-rich enteral nutrition prevented intestinal barrier dysfunction in a model of septic shock by activating the CAP through CCK receptors; this effect was prevented by vagotomy and CCK or nicotinic receptor antagonists (Luyer et al., 2005; de Haan et al., 2010).

Endotoxemia following intraperitoneal LPS induces intestinal barrier dysfunction with a decreased expression of occludin and zonula occludens 1 with a disruption of tight junction and increase of intestinal permeability. These effects were prevented by VNS and dampened by pretreatment of the animals with an α7nAChR antagonist before VNS (Zhou et al., 2013). VNS also prevents burn-induced intestinal permeability by improving tight junction protein expression of occludin (Costantini et al., 2010; Krzyzaniak et al., 2011). The α7nAChR protects against burn-induced gut barrier injury by preventing the decreased expression and altered localization of occludin and zonula occludens-1 (ZO-1) (Costantini et al., 2012). The mechanism through which vagal efferents protect barrier epithelium is not well-known. One of the mechanisms could be the connection of the VN with the enteric nervous system which communicates with enteric glia cells through nicotinic cholinergic signaling (Cheadle et al., 2014). These glia cells preserve epithelial barrier against intestinal bacteria insult by increasing the expression of tight junction proteins such as occludin and ZO-1 through the secretion of S-nitrosoglutathione (Yu and Li, 2014). However, a recent study has shown that enteric glia is not required for maintenance of the intestinal epithelium (Rao et al., 2017). Consequently, vagal activity provides a protective function to the intestinal epithelial barrier and a low vagal activity makes intestinal epithelium more permeable thus promoting systemic inflammation and chronic disease.

There are presently no published data regarding the effect of vagal stimulation either chemically or through VNS on the gut microbiota but based on its effect on intestinal permeability and local immunity (Meregnani et al., 2011), we can hypothesize that the VN could modulate gut microbiota composition that depends on these two factors (Karl et al., 2017).

## Stress, vagus nerve, and the microbiota

The effect of stress on the GI tract is well-known (Taché and Bonaz, 2007). Stress, through its neuromediators, corticotrophin-releasing factor (CRF) and its related peptide urocortin(s), acting on their G protein coupled CRF (1 and 2) receptors located in the brain and the GI tract, increases intestinal permeability and modifies the gut microbiota (Taché et al., 2018); these two factors are involved in the pathophysiology of IBS and IBD. CRF2 receptors are involved in the perturbation of intestinal permeability (Ducarouge et al., 2017). CRF and urocortin are released by mastocytes of the lamina propria which have CRF1-2 receptors, activation of which releases cytokines and other pro-inflammatory mediators by mastocytes (Theoharides and Cochrane, 2004; Theoharides et al., 2004). CRF also increases intestinal permeability via mast cell release of tumor necrosis factor (TNF)-α and proteases (Overman et al., 2012). Targeting CRF receptors by selective antagonists to inhibit mast cell activation is a therapeutic option for chronic inflammatory disorders exacerbated by stress. Chronic early life stress induces dysbiosis in rats via modifications of intestinal permeability, which may later sensitize adult rats to visceral hypersensitivity (Moussaoui et al., 2017). Classically, stress inhibits the VN and stimulates the sympathetic nervous system through autonomic-related projection neurons of the PVH to the dorsal motor nucleus of the VN and sympathetic pre-ganglionic neurons of the spinal cord (Taché and Bonaz, 2007; Wood and Woods, 2007). Since the VN has anti-inflammatory properties through its afferent and efferent fibers (Bonaz et al., 2016a), stress has thus pro-inflammatory properties. A single acute stress induces a prolonged increase of pro-inflammatory cytokines after the end of stress exposition (Marsland et al., 2017), at the moment of the recovery period which is a critical period since it corresponds to the parasympathetic rebound. Exposition to multiple repeated stressors counteracts the parasympathetic tone recovery, favoring an allostatic load (McEwen, 2008) thus blunting the anti-inflammatory regulatory action of the VN. Stress could counterbalance the overall protective effect of the VN on epithelial barrier and thus favor dysbiosis by disrupting epithelial homeostasis.

## Conclusion

The role of the VN in microbiota-brain communication is now well-established. A reduction in vagal tone reflecting dysautonomia has been shown in IBS and IBD (Pellissier et al., 2010, 2014) characterized by a leaky gut and dysbiosis (Bonaz et al., 2016b). Consequently, monitoring vagal tone would be an interesting marker of the microbiota-gut-brain axis. Relevant electrophysiological data could be then considered as a part of the—omes, and should be integrated in a converging combined approach to decipher complex IBD and IBS pathophysiology (de Souza et al., 2017). Moreover, monitoring and targeting vagal tone through VNS, microbiota modulation (using prebiotics, probiotics, fecal microbiota transplantation, diet…), drugs targeting the cholinergic system and/or complementary medicine (hypnosis, meditation), cognitive behavioral therapies, deep breathing, and moderate and sustainable physical activity would be of interest to restore a homeostatic microbiota-gut-brain axis.

## Author contributions

BB wrote the first draft of the manuscript; TB and SP provided critical feedback to improve it; TB drew the figures.

### Conflict of interest statement

The authors declare that the research was conducted in the absence of any commercial or financial relationships that could be construed as a potential conflict of interest.
